# Differential characteristics among asymptomatic and symptomatic meibomian gland dysfunction and those with dry eye

**DOI:** 10.1186/s12886-023-02878-5

**Published:** 2023-04-11

**Authors:** Yi-Ran Chiou, Pei-Yu Lin, Yu-Bai Chou, Po-Wei Huang, Nai-Wen Fan

**Affiliations:** 1 Department of Ophthalmology, Taipei Veterans General Hospital, Taipei, Taiwan; 2 Faculty of Medicine, School of Medicine, National Yang Ming Chiao Tung University, Hsinchu, 30010 Taiwan; 3 Department of Radiation Oncology, Shuang Ho Hospital, Taipei Medical University, New Taipei City, Taiwan

**Keywords:** Dry eye disease, Interferometry, Lipid layer thickness, Meibomian gland dysfunction, Ocular surface parameters

## Abstract

**Purpose:**

To identify the characteristics of asymptomatic meibomian gland dysfunction (MGD), symptomatic MGD, and MGD coexisting with dry eye disease (DED).

**Methods:**

This cross sectional study enrolled a total of 153 eyes of 87 MGD patients. Participants filled in ocular surface disease index (OSDI) questionnaires. Age, gender, Schirmer’s test, meibomian gland (MG) related parameters, lipid layer thickness (LLT) and blinking were compared among patients with asymptomatic MGD, symptomatic MGD, and MGD with DED. Multivariate regression was used to analyze the significant factor of DED in MGD. Spearman’s rank correlation analysis was used to evaluate the association between the significant factors and MG function.

**Results:**

There was no difference in age, Schirmer’s test, lid changes, MG secretion, and MG morphology among three groups. The OSDI of asymptomatic MGD, symptomatic MGD and MGD coexisting with DED were 8.5 ± 2.9, 28.5 ± 12.8 and 27.9 ± 10.5, respectively. Patients with MGD coexisting with DED exhibited more frequent eye blinking than that of patients with asymptomatic MGD (8.1 ± 4.1 vs. 6.1 ± 3.5 blinks/20 sec, *P* = 0.022), and reduced LLT than that of patients with asymptomatic MGD (68.6 ± 17.2 vs. 77.6 ± 14.5 nm, *P* = 0.010) and symptomatic MGD (78.0 ± 17.1 nm, *P* = 0.015). Multivariate analysis identified LLT (per nm, OR = 0.96, 95% CI = 0.93—0.99, *P* = 0.002) as a significant factor associated with DED development in MGD. The number of expressible MG was positively correlated with LLT (Spearman’s correlation coefficient = 0.299, *P* = 0.016) but negatively correlated with the number of blinking (Spearman’s correlation coefficient = -0.298, *P* = 0.016) in MGD patients with DED, and these findings were not identified in those without DED.

**Conclusions:**

Asymptomatic MGD, symptomatic MGD, and MGD coexisting with DED share similar characteristics, including meibum secretion and morphology, but MGD patients coexisting with DED exhibited significantly reduced LLT.

## Introduction

Meibomian glands (MG) are modified sebaceous glands located in the upper and lower tarsus, and the glandular secretion, meibum, constitutes the essential lipid component of the tear film, which stabilizes the tear film at eye opening [1]. Dysfunction of MG (MGD) is commonly characterized by terminal duct obstruction and/or qualitative/quantitative changes in the glandular secretion, and potentially results in tear film instability, hyperosmolarity, ocular surface inflammation, and ocular surface damage. MGD has been considered an important public health issue in recent years because of its high prevalence, with reports up to 70% by population-based studies, especially more common in Asian population [2]. In contrast to dry eye disease (DED), presence of ocular symptoms is not mandatorily included in the diagnostic criteria of MGD in clinical studies [2–4]. Therefore, the diagnosis of MGD can be undoubtedly made by objective clinical signs, such as lid margin telangiectasia, plugging of orifice, and altered meibum expression upon firm digital expression [5, 6]. Consequently, with such definition, researcher found that MGD patients are largely asymptomatic, in which less than one-third of MGD patients have relevant ocular symptoms [4, 5].

The ocular symptoms of MGD are similar to those of DED, such as sore eyes, ocular fatigue, heavy sensation, dryness and redness [4]. A recent population-based study conducted in Japan analyzed the factors associated with the prevalence of MGD and DED, showing that these two diseases are different in gender prevalence, tear film stability and ocular surface presentations [4]. The difference observed in the two diseases was also reported in other studies. The underlying basis of DED-related symptoms or disease severity is highly linked to tear film stability [3, 5], but this causal relationship is not unequivocally found in MGD patients because the symptoms of MGD may also come from the inflammation of posterior blepharitis [7, 8]. Symptomatic MGD patients may even exhibit a better tear film stability and less ocular surface fluorescein staining than asymptomatic MGD patients [5]. Therefore, the pathogenesis is different among asymptomatic MGD, symptomatic MGD, and the MGD patients co-exhibiting DED, who also lose tear film homeostasis. Unfortunately, MGD coexisting with DED accounts for more than one-third of MGD patients [4, 9].

Collectively, asymptomatic MGD, symptomatic MGD, and MGD coexisting with DED are substantially at different stages of the disease. In literature, the preponderance studies which investigate the characteristics of MGD-related DED commonly exclude the patients with aqueous deficiency based on the result of Schirmer’s test [10–12]. However, aqueous deficient DED and evaporative DED are not mutually exclusive. The most recent Dry Eye WorkShop (DEWS II) report showed that it is difficult to clearly distinguish aqueous deficient DED and evaporative DED, and thus advocated a continuum classification, emphasizing that overlap between these two subtypes are highly common [13, 14]. Therefore, research investigating MGD patients who exhibit evidence of DED, rather than strictly being restricted to investigate evaporative DED alone, is required in the real world. Herein, the current cross sectional study conducted in Taiwan was designed to group Asian MGD patients according to the DED definition updated by Asia Dry Eye Society [15], to evaluate the characteristics of asymptomatic MGD, symptomatic MGD, and MGD coexisting with DED, and to further identify the factor associated with DED development in MGD patients.

## Methods

### Subjects

This cross sectional study enrolled MGD patients between December 2014 and March 2015. This study was approved by the Institutional Review Boards of Taipei Veterans General Hospital and adhered to the tenets of the Declaration of Helsinki for human studies. Written informed consent was obtained from all participants. Inclusion criteria included a diagnosis of MGD. Exclusion criteria included a presence of uncontrolled systemic diseases, concurrent active ocular surface inflammation or infection within 1 month, eyelid disorders causing exposure of the ocular surface, and prior corneal transplantation surgery or lacrimal surgery. All participants were requested not to use eye ointment in the last 24 h, eye drops, contact lens, or swim in the last 12 h. On the day of examinations, participants were informed of not using eyeliner or makeup as well as avoiding eye rubbing within 2 h before examination.

### Questionnaires

Ocular Surface Disease Index (OSDI) symptom questionnaire, which is a widely used questionnaire for dry eye evaluation, was used in this study, and assisted by a well-trained investigator. The score ranges from 0 to 100 (most severe symptom) [16].

### Examinations

Lipid layer thickness (LLT) and blinking pattern within 20 s were evaluated using LipiView interferometer (Johnson & Johnson, New Brunswick, New Jersey), and all participants were told to blink normally during whole examinations. To get a reliable LLT, the same examination was performed again after participants rested for 10 min. The average of LLT was used for analysis in this study. Then, two corneal specialists (P.-Y. Lin and N.-W. Fan) assessed tear film break-up time (TBUT), Schirmer’s test without anesthetic, absence or presence of lid telangiectasia, absence or presence of anterior displacement of mucocutaneous junction over lateral third, middle, and medial third of lower eyelids and each area was scored 0 for absence and 1 point for presence. The meibum expressibility over whole lower eyelids in response to moderate digital pressure was evaluated and the total number of expressible glands was counted. Meibum quality was scored as follows: 0 = clear liquid, 1 = cloudy fluid, 2 = cloudy with particulate fluid, 3 = toothpaste-like meibum, and 4 = total atrophic meibum. Meiboscore was determined by the loss of meibomian gland detected by transillumination through everted eyelids, scored from grade 0 through grade 3 (0 = no loss of meibomian gland, 1 = less than 25% loss, 2 = 25–50% loss, 3 = 50–75% loss, 4 = more than 75% loss) [17].

### Definition of MGD and DED

MGD was defined as (1) no expression or increased viscosity of meibum secretion in response to moderate digital expression, and (2) presence of lid abnormality, such as lid margin telangiectasia, orifice plugging, or displacement of the mucocutaneous junction [4, 5]. The definition of DED in this study is adherent to the consensus report by Asia Dry Eye Society, which defines dry eye disease based on tear film-oriented concept, adopting a cutoff value of less than 5 s of TBUT along with presence of DE symptoms for the diagnosis of all subtypes of dry eye disease [15]. Thus, in our study, OSDI was used to quantify patients’ symptoms, defining OSDI score ≥ 13 as presence of DE or MGD symptoms [18, 19]. Therefore, subjects were further grouped as followings: asymptomatic MGD (Group A, OSDI < 13), symptomatic MGD (Group B, OSDI ≥ 13 and TBUT ≥ 5), and MGD with DED (Group C, OSDI ≥ 13 and TBUT < 5).

### Statistical analysis

Data were analyzed using statistical software IBM SPSS Statistics for Windows Version 25.0 (IBM Corp., Armonk, N.Y., USA). The data were presented as means (standard deviations) for continuous variables and as counts (percentages) for categorical variables. Testing for normal distribution was performed using Shapiro-Wilk test. For comparison of multiple groups with normally distributed variables, one-way analysis of variance (ANOVA) was used followed by post hoc test using Tukey-Kramer method. For comparison of multiple groups with non-normally distributed variables, Kruskal-Wallis test was used followed by post hoc test using Bonferroni correction.

To evaluate the factors associated with MGD coexisting with DED (Group C), univariate and multivariate logistic regression were used. For the multivariate model, all variables of interest were selected regardless of the P value due to the multifactorial etiology contributing to DED. If collinearity is shown between selected variables, only one would be chosen for subsequent multivariate model. The receiver operating characteristic (ROC) analysis was used to assess the associated factors for determining DED related to MGD. In addition, Spearman’s rank correlation analysis was used to evaluate the association between the significant factors detected in multivariate analysis and other ocular parameters. A P value less than 0.05 was regarded as statistically significant.

## Results

**Fig. 1:**
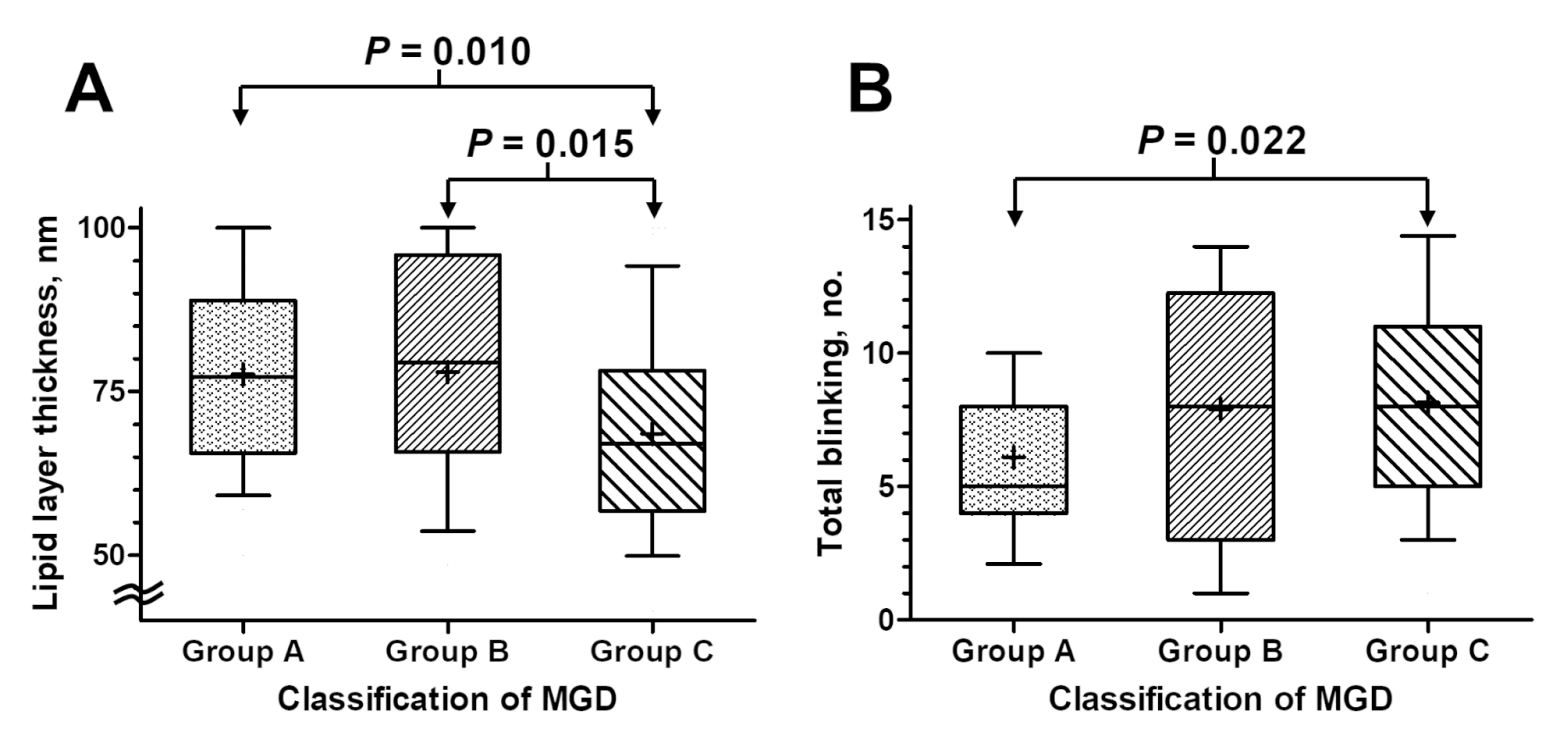
Box-and-whisker graphs showing clinical examinations (mean is presented as a cross symbol within the box, the 10th and 90th percentile value is presented as whiskers) of (**A**) lipid layer thickness and (**B**) total blinking in the three groups

A total of 153 eyes of 87 patients (35 males, 52 females) aged 64.0 ± 12.6 years were diagnosed with MGD, of whom 50 eyes had asymptomatic MGD (Group A), 38 eyes had significant ocular surface symptoms (Group B); and 65 eyes had DED (Group C). The baseline characteristics and ocular findings among three groups were shown in Table 1. Symptomatic MGD had significant longer TBUT (6.2 ± 1.3 s) than asymptomatic MGD (5.5 ± 3.7 s, *P* = 0.014). Patients with MGD and DED had reduced LLT as compared to other two groups (Group A, *P* = 0.010; Group B, *P* = 0.015), as shown in Fig. 1A. In addition, MGD with DED (Group C) exhibited more frequent eye blinking as compared to asymptomatic MGD (Group A), illustrated in Fig. 1B (*P* = 0.022). There was no significant difference among 3 groups in gender prevalence, the value of Schirmer’s 1 test, number of patients with Schirmer’s 1 test ≤ 5 mm, pattern of blinking, telangiectasia, severity of mucocutaneous junction displacement, meibum expressibility, meiboscore, and grading of meibum quality.

**Table 1:** Characteristics and ocular findings of the studied eyes

	Asymptomatic MGD (Group A)	Symptomatic MGD (Group B)	MGD with DED (Group C)	
	(n = 50 eyes)	(n = 38 eyes)	(n = 65 eyes)	*P* Value
Age (years)	64.0 ± 14.7	64.6 ± 11.2	64.0 ± 11.7	0.952
Female eyes, no. (%)	30 (60.0)	19 (50.0)	47 (72.3)	0.070
OSDI (score)	8.5 ± 2.9	28.5 ± 12.8	27.9 ± 10.5	**< 0.001** ^*‡^
TBUT (sec)	5.5 ± 3.7	6.2 ± 1.3	3.0 ± 0.9	**< 0.001** ^*†‡^
Schirmer’s 1 test (mm)	12.9 ± 10.8	14.0 ± 8.4	16.1 ± 10.9	0.377
Schirmer’s 1 test ≤ 5 mm, no. (%)	14 (28.0)	6 (15.8)	14 (21.5)	0.390
LLT (nm)	77.6 ± 14.5	78.0 ± 17.1	68.6 ± 17.2	**0.003** ^†‡^
Total blinking (number/20 sec)	6.1 ± 3.5	7.9 ± 5.2	8.1 ± 4.1	**0.025** ^‡^
Complete blinking (number/20 sec)	2.6 ± 2.7	4.4 ± 5.4	4.2 ± 4.2	0.239
Incomplete blinking (number/20 sec)	3.5 ± 3.2	3.5 ± 4.3	3.9 ± 3.8	0.415
Telangiectasia, no. (%)	38 (76.0)	28 (73.7)	45 (69.2)	0.774
Meibum expressibility (number/LE)	10.5 ± 3.9	11.7 ± 4.2	10.8 ± 4.9	0.458
Meibum quality grading, no. (%)				0.957
Grade 0	2 (4.0)	3 (7.9)	5 (7.8)	
Grade 1	36 (72.0)	25 (65.8)	45 (67.7)	
Grade 2	11 (22.0)	9 (23.7)	10 (15.6)	
Grade 3	1 (2.0)	0 (0.0)	3 (4.7)	
Grade 4	0 (0.0)	1 (2.6)	2 (3.1)	
Meiboscore, no. (%)				0.795
Grade 0	1 (2.0)	3 (7.9)	2 (3.1)	
Grade 1	21 (42.0)	15 (39.5)	29 (44.6)	
Grade 2	21 (42.0)	11 (28.9)	16 (25.0)	
Grade 3	7 (14.0)	8 (21.1)	13 (20.3)	
Grade 4	0 (0.0)	1 (2.6)	5 (7.8)	
MCJ displacement grading, no. (%)				0.927
Grade 0	6 (12.0)	3 (7.9)	4 (6.2)	
Grade 1	30 (60.0)	25 (65.8)	47 (72.3)	
Grade 2	12 (24.0)	5 (13.2)	6 (9.2)	
Grade 3	2 (4.0)	5 (13.2)	8 (12.3)	

MGD = meibomian gland disease; DED = dry eye disease; OSDI = Ocular Surface Disease Index; TBUT = tear film break-up time; LLT = lipid layer thickness; LE = lower eyelid; MCJ = mucocutaneous junction

^*^Group A vs. Group B significant

^†^Group B vs. Group C significant

^‡^Group A vs. Group C significant

To study the factors associated with coexistence of MGD with DED, we analyzed parameters between MGD with DED (Group C) and those without DED (Group A + B). In univariate and multivariate logistic regression model, LLT was statistically significant associated with coexistence of MGD with DED (*P* = 0.001 and 0.002 respectively) (Table 2).

**Table 2:** Univariate and multivariate logistic regression models analyzing the factors associated with meibomian gland disease with dry eye disease

	Univariate			Multivariate			
	Odds ratio	95% CI	*P* value		Odds ratio	95% CI	*P* value
Schirmer’s 1 test (mm)	1.03	0.99—1.06	0.117		1.01	0.98—1.06	0.128
LLT (nm)	0.97	0.95—0.99	**0.001**		0.96	0.93—0.99	**0.002**
Total blinking (number/20 seconds)	1.07	0.99—1.16	0.073		1.09	0.99—1.21	0.093
Incomplete blinking (number/20 seconds)	1.03	0.95—1.13	0.461		1.01	0.90—1.14	0.831
Telangiectasia	0.77	0.38—1.58	0.482		0.89	0.38—2.10	0.787
Meibum expressibility	0.99	0.92—1.06	0.759		1.05	0.95—1.14	0.340

CI = confidence interval; LLT = lipid layer thickness

**Fig. 2:**
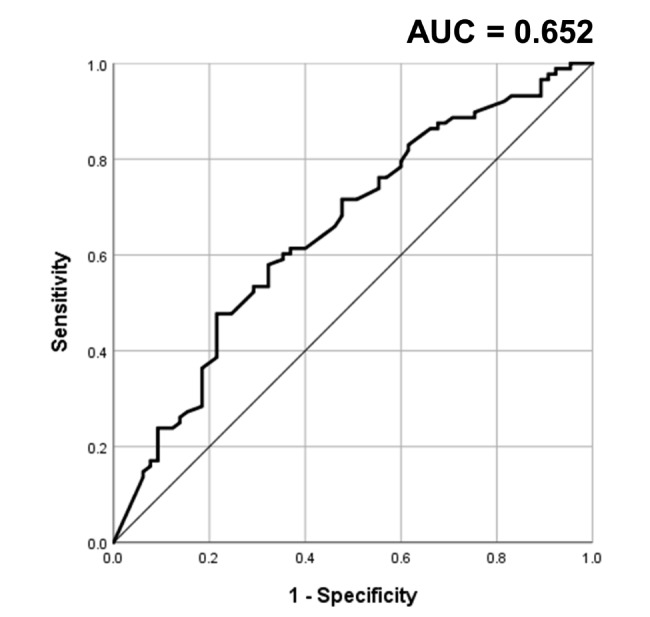
The receiver operating characteristics (ROC) curve for lipid layer thickness of the eyes with coexistence of meibomian gland dysfunction and dry eye disease. AUC, area under the curve

Next, ROC analysis was used for an optimal cut-off value of LLT for the likelihood of coexistence of MGD with DED. The result showed that for a cut-off value of LLT ≤ 73 nm, the sensitivity was 60.2% and specificity was 64.6% (χ2 test for the 4-fold tables P = 0.001). Figure 2 shows the area under the curve (AUC) equals to 0.652.

**Fig. 3:**
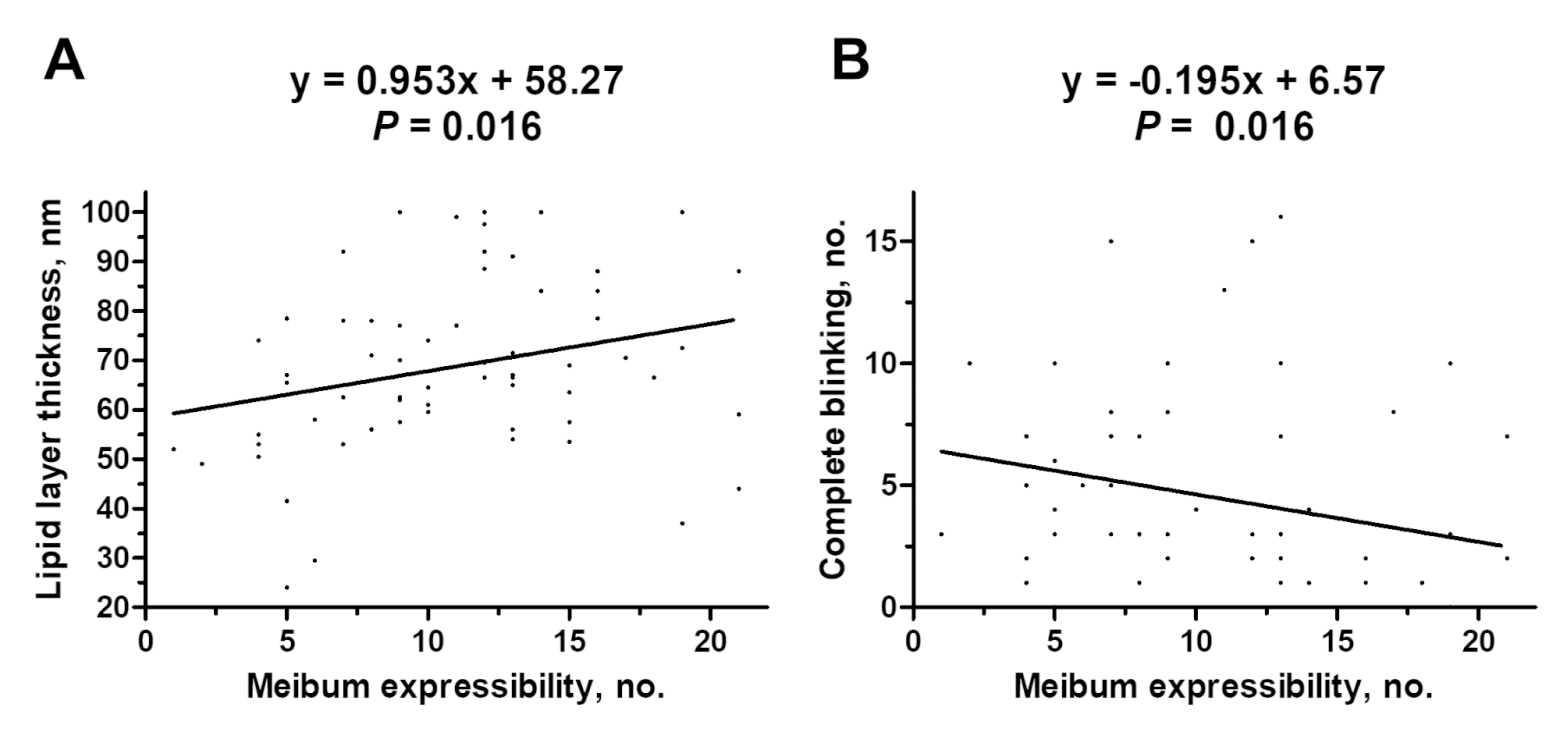
Correlations between (**A**) meibum expressibility and lipid layer thickness or (**B**) meibum expressibility and complete blinking in the eyes with coexistence of meibomian gland dysfunction and dry eye disease

Spearman correlations were performed to study the correlation between age, meibum expressibility, and other ocular parameters which were listed in Table 1. The significant results were shown in Table 3. Age had a negative correlation with either meibum expressibility or total blinking in eyes with asymptomatic MGD (Group A; Spearman’s correlation coefficient = -0.281 and − 0.311, *P* = 0.048 and 0.028, respectively), but the correlation was not found in symptomatic MGD (Group B) or those with coexistence of MGD and DED (Group C). There was no correlation between meibum expressibility and LLT or complete blinking in MGD patients without DED. In contrast, in MGD patients with DED, meibum expressibility was correlated with LLT (Spearman’s correlation coefficient = 0.299, *P* = 0.016) and negatively correlated with complete blinking (Spearman’s correlation coefficient = -0.298, *P* = 0.016). The scattered plots of data were shown in Fig. 3A and B, respectively.

**Table 3:** Correlations between age, meibum expressibility, and other parameters among three groups

		Asymptomatic MGD	Symptomatic MGD	MGD with DED
		(Group A)	(Group B)	(Group C)
**Age**				
Age and meibum expressibility	Spearman correlation	-0.281	-0.064	-0.053
	*P* value	**0.048**	0.705	0.677
Age and total blinking	Spearman correlation	-0.311	0.103	-0.108
	*P* value	**0.028**	0.546	0.397
**Meibum expressibility**				
Meibum expressibility and LLT	Spearman correlation	-0.273	0.263	0.299
	*P* value	0.055	0.111	**0.016**
Meibum expressibility and complete blinking	Spearman correlation	0.016	-0.246	-0.298
	*P* value	0.913	0.137	**0.016**

LLT = lipid layer thickness

## Discussion

The most important clinical parameters for the assessment of MGD include lid margin changes and reduced quantity (obstruction) or quality of meibum [20]. In our study, none of them was different among asymptomatic MGD, symptomatic MGD, and MGD with DED. We found that eyes with coexistence of MGD and DED were characterized by reduced LLT and more frequent eye blinking as compared to asymptomatic MGD. Among all the MG-related parameters, LLT is the only factor to be able to distinguish MGD patients with DED from those without, with a cut-off value LLT ≤ 73 nm. In addition, LLT is positively correlated with meibum expressibility in eyes with coexistence of MGD and DED, but not in MGD without DED.

The role of LLT in MGD evaluation remains controversial [21–23]. In our study, LLT was similar between symptomatic MGD and asymptomatic MGD, but was significantly reduced in MGD patients with DED. In our cohort, LLT of MGD patients without DED is comparable to the normal range reported by other studies [19, 20, 24]. Thus, our study suggests that LLT should not be weighed as a standard measurement of the function of MG, but rather as a useful screening tool for detecting DED in MGD patients. Given that meibum expressibility, meibum quality, and MG dropout (evaluated using meiboscore) are similar between MGD patients with and without DED, it also suggests LLT is not exclusively determined by these MG parameters, but rather, other factors, such as compensatory mechanism should be taken into account. Our study shows LLT is correlated with the number of expressible MG in MGD patients with DED, but not in MGD patients without DED. Hence, it suggests MGD patients without DED may still have functional MGs which can compensatorily increase the secretion to maintain a level of lipid layer quantity and ocular surface health, but MGD patients with DED may have impaired compensatory mechanism, resulting in development of DED [8, 25].

In our cohort, MGD patients with DED exhibit higher frequencies of total blinking, up to 8 blinks/20 seconds, which is more than two-fold normal blinking rate 10–16 blinks/min (3–5 blinks/20 seconds) [26, 27], and the complete blinking rate is negatively correlated with the number of expressible glands. There are three possible reasons to explain the finding of increased blinking in MGD patients with DED. First, increased total blinking may be a compensatory effect to comfort the DE-related ocular discomfort. Second, DED patients may have altered corneal nerves. When eye blinking is recorded by Lipiview, a light originated from Lipiview in a dim room may induce more reflex blinking in patients with trigeminal nerve sensitization [28]. Third, increased total blinking may decrease the force of each blinking and decrease the ability to augment the expression of lipids from MG, and thus results in reduced lipid layer in MGD patients with DED [29, 30].

In correlation analysis of age, LLT, meibum expressibility, and other parameters, different results were found in the subgroup analysis. Age was negatively correlated with meibum expressibility and blinking in patients with asymptomatic MGD, but not in symptomatic MGD or MGD with DED. In contrast, meibum expressibility was positively correlated with LLT in MGD patients with DED, but not in patients with asymptomatic MGD or symptomatic MGD. The different results among subgroups may indicate that the disease pathogenesis was different. Previous studies suggested the meibum secretion of MG and the number of voluntary blinks substantially reduced with age [17, 31–33] Thus, asymptomatic patients in our cohort who had reduced meibum expressibility and decreased blinking with age may represent the category of natural change of MGD with age, which does not cause significant ocular symptoms. The dissociation between age and meibum expressibility in other two groups suggests the pathogenesis of these two diseases are involved by multiple factors, such as meibum chain branching, in addition to aging [34]. The ocular surface symptoms may induce compensatory blinking and thus mask the age related blinking reduction in symptomatic MGD and MGD with DED.

Some limitations exist in this study. This hospital-based study lacks age- and sex-matched normal control and patients who come to hospital are largely symptomatic. In our study, the number of MGD patients with symptoms or unstable tear film was larger than that of asymptomatic MGD, in contrast to the population based study in China, where asymptomatic MGD is found to be 3 times more common than symptomatic MGD [35]. Moreover, our cohort lacks severe dry eye patients. The reason might be that we excluded patients with active ocular surface inflammation, such as Sjogren’s syndrome and ocular graft-versus-host disease. Further studies are warranted to identify the characteristics of MGD patients with more severe DED symptoms.

The common clinical parameters to assess disease severity of MGD include meibum expressibility, meibum quality, lid margin changes, and MG dropout. In our cohort, none of these general clinical parameters is able to distinguish asymptomatic MGD, symptomatic MGD, and MGD coexisting with DED. LLT is the only factor to be associated with MGD with DED. LLT represents the quantity of meibum, rather than the quality of meibum (altered lipid components, or altered lipid dynamics) [24], and the influential factors of LLT is not limited to the MG function, but also affected by other factors, such as blinking and compensatory mechanism [36]. Therefore, the value of LLT has not been validated for the diagnosis of MGD [23]. Our study shows that MGD patients with DED had reduced LLT, suggesting that these DED patients may have impaired compensatory mechanism for dysfunctional MG, resulting in reduced overall quantity of meibum on the ocular surface. Thus, LLT could be a useful screening tool for identification of DED in MGD patients, and further studies are warranted to untangle the pathogenesis contributing to DED in MGD patients.

## Data Availability

The dataset generated during and/or analysed during the current study are available from the corresponding author on reasonable request.

## Abbreviations

MG: Meibomian glands
MGD: Meibomain gland dysfunction
DED: Dry eye disease
DE: Dry eye
OSDI: Ocular Surface Disease Index
LLT: Lipid layer thickness
TBUT: Tear film break-up time
